# Predicting response to CCRT for esophageal squamous carcinoma by a radiomics-clinical SHAP model

**DOI:** 10.1186/s12880-023-01089-0

**Published:** 2023-10-02

**Authors:** Xu Cheng, Yuxin Zhang, Min Zhu, Ruixia Sun, Lingling Liu, Xueling Li

**Affiliations:** 1 Hefei Cancer Hospital, Chinese Academy of Sciences, Hefei, 230031 P.R. China; 2 Anhui Province Key Laboratory of Medical Physics and Technology, Institute of Health and Medical Technology, Hefei Institutes of Physical Science, Chinese Academy of Sciences, Hefei, 230031 P.R. China; 3 School of Biomedical Engineering, Anhui Medical University, Hefei, 230032 China; 4 School of Mathematics and Computer Science, Tongling University, Tongling, China

**Keywords:** Esophageal squamous carcinoma, Concurrent chemoradiotherapy, Radiomics, SHAP model

## Abstract

**Background:**

Radical concurrent chemoradiotherapy (CCRT) is frequently used as the first-line treatment for patients with locally advanced esophageal cancer. Unfortunately, some patients respond poorly. To predict response to radical concurrent chemoradiotherapy in pre-treatment patients with esophageal squamous carcinoma (ESCC), and compare the predicting efficacies of radiomics features of primary tumor with or without regional lymph nodes, we developed a radiomics-clinical model based on the positioning CT images. Finally, SHapley Additive exPlanation (SHAP) was used to explain the models.

**Methods:**

This retrospective study enrolled 105 patients with medically inoperable and/or unresectable ESCC who underwent radical concurrent chemoradiotherapy (CCRT) between October 2018 and May 2023. Patients were classified into responder and non-responder groups with RECIST standards. The 11 recently admitted patients were chosen as the validation set, previously admitted patients were randomly split into the training set (*n* = 70) and the testing set (*n* = 24). Primary tumor site (GTV), the primary tumor and the uninvolved lymph nodes at risk of microscopic disease (CTV) were identified as Regions of Interests (ROIs). 1762 radiomics features from GTV and CTV were respectively extracted and then filtered by statistical differential analysis and Least Absolute Shrinkage and Selection Operator (LASSO). The filtered radiomics features combined with 13 clinical features were further filtered with Mutual Information (MI) algorithm. Based on the filtered features, we developed five models (Clinical Model, GTV Model, GTV-Clinical Model, CTV Model, and CTV-Clinical Model) using the random forest algorithm and evaluated for their accuracy, precision, recall, F1-Score and AUC. Finally, SHAP algorithm was adopted for model interpretation to achieve transparency and utilizability.

**Results:**

The GTV-Clinical model achieves an AUC of 0.82 with a 95% confidence interval (CI) of 0.76–0.99 on testing set and an AUC of 0.97 with a 95% confidence interval (CI) of 0.84–1.0 on validation set, which are significantly higher than those of other models in predicting ESCC response to CCRT. The SHAP force map provides an integrated view of the impact of each feature on individual patients, while the SHAP summary plots indicate that radiomics features have a greater influence on model prediction than clinical factors in our model.

**Conclusion:**

GTV-Clinical model based on texture features and the maximum diameter of lesion (MDL) may assist clinicians in pre-treatment predicting ESCC response to CCRT.

## Introduction

In 2020, one out of 18 cancer deaths will be due to esophageal cancer, the seventh most common cancer in the world [1]. The standard treatment for esophageal cancer is surgery and radiotherapy plays an important part of that. The National Comprehensive Cancer Network (NCCN) guidelines recommend radical concurrent chemoradiotherapy (CCRT) as the first-line treatment for patients [2]. CCRT improves survival over radiotherapy alone, especially in patients with unresectable disease or medically unfit condition for surgical intervention, and CCRT is now the standard treatment for patients suffering from locally advanced esophageal cancer [3–5]. However, in patients with esophageal cancer, the overall response rate to radical CCRT is between 53.3% and 98.3% [6]. If the first-line radical CCRT treatment is unsuccessful, ineffective CCRT will definitely delay and interrupt otherwise potential effective treatment. Furthermore, CCRT may lead to side effects [7, 8]. CCRT for esophageal cancer can aggravate bone marrow suppression, and about 0 ~ 94.7% patients may develop hematological toxicity during radiotherapy for esophageal cancer [9]. Fatal radiotherapy complications such as esophageal fistula and radiation pneumonia will occur in 5.3 ~ 24.1% [10] and 0.6 ~ 6.7% of patients [9], respectively. Therefore, predicting the response to CCRT prior to treatment initiation may help us determine if we choose CCRT as the first-line therapy and guide individualized dosing based on response prediction results for CCRT-sensitive patients and thus help patients gain the greatest benefits.

In clinical practice, determining a patient's response to CCRT for ESCC is typically administered during or after therapy based on tumor biopsy and imaging tests, neither of which are helpful in predicting response and prognostics before treatment. Post-treatment CT image is used in clinical practice to assess the efficacy of CCRT, which is non-invasive but remains a lagging indicator of treatment response. Moreover, evaluated morphological changes are limited to those that can be observed with the naked eye, and the assessment is highly subjective. As a result, accurate methods for predicting CCRT response merit further investigation.

Radiomics is a relatively new research field that methodically handles the vast amount of imaging data in radiology and its association with cancer clinical stages and outcomes. It can detect features that reflect intra- and inter-tumoral heterogeneity., which was reported to be associated with sensitivity to different treatment modalities including chemotherapy, radiotherapy and other treatments [11–14]. It has been demonstrated in a number of studies that CT image radiomics analysis can be used to precisely anticipate an individual's survival in esophageal cancer [15–18]. In radiation oncology, patients are treated with radiotherapy based on the outline of the target area, i.e., the outlines of GTV (Gross Tumor Volume) and CTV (the primary tumor and the uninvolved lymph nodes at risk of microscopic disease),which are very critical in the outlining of the target area. However, these studies focused only on the radiomics features of primary tumor. Few studies have used radiomics features to compare GTV versus CTV in predicting CCRT response in ESCC patients. Recently, the prognostic value of primary tumor and metastatic lymph node radiomics in overall survival of ESCC patients has been described [19]. However, the model did not specify the treatment modality, its conclusion may not apply to the CCRT response prediction, and thus have limited application in decision making for precision treatment.

Taking these into consideration, we developed multiple radiomics models for predicting response to CCRT for ESCC patients and compared the significance of GTV and CTV from the pre-treatment positioning enhanced CT images. The CCRT response prediction model was evaluated and finally explained by SHAP for its interpretability and transparency. Our GTV-Clinical model has potential to stratify patients in pre-treatment.

## Materials and methods

### Patient inclusion

One hundred and five consecutive patients with ESCC (treated from January 2018 through May 2023) were retrospectively enrolled in accordance with the inclusion criteria listed below: (1) histologically (biopsy) proven esophageal squamous carcinoma and stage II to IVA disease (based on the 8th edition of the American Joint Committee on Cancer [20]). (2) a performance status of 0 to 1 for the Eastern Cooperative Oncology Group; (3) adequate bone marrow, hepatic, and kidney function; (4) received concurrent chemoradiotherapy and had response information. And the exclusion criteria as follows: (1) patients with tracheoesophageal fistula; (2) a history of interstitial pneumonia; (3) active infected persons; (4) patients with severe cardiovascular disease, malignant pleural effusion or other concomitant cancers; (5) patients undergoing treatment with other investigational drugs or other clinical trials; (6) allergic to this product or its auxiliary materials; (7) suffering from mental or nervous system diseases and unable to cooperate; (8) the time of the final enhancement localization CT was not more than one week before the first radiotherapy for patients with esophageal cancer. The validation group consisted of eleven recent admitted patients, 25% of the remaining patients were divided into testing groups using computer random number generation [*n* = 24, mean age: 68.08 ± 8.14, range: 44–80 years] and the other patients were divided into the training set [*n* = 70, mean age: 67.02 ± 8.68, range: 41–82 years].

All patients received standard radical CCRT in accordance with NCCN guidelines [2]. The total dose of radiotherapy was between 50.4-60 Gy, and the frequency of fractionation was 28–30 times. The concurrent chemotherapy regimen included paclitaxel, platinum and 5-FU.

### CT imaging

In the process of free breathing, all patients were required to undergo the positioning CT scanning before radical CCRT. All patients got contrast-enhanced chest CT (CT Simulation Position, Brilliance CT Big Bore, Philips Medical Systems (Cleveland) lnc, USA) before getting therapy. For the purpose of extracting image features, arterial-phase CT scans were acquired. The scanning layer thickness was 5 mm. The image data obtained from the same CT scanner were input into the CT simulation workstation through the network to delineate the target area.

### Delineations and volume of interest segmentation

Three-dimensional volume of interest corresponding to the GTV and CTV on each positioning CT were manually delineated slice-by-slice with Elekta Monaco planning system V3.8.0 (Monaco) by a qualified radiologist (five years’ experience in esophageal imaging) and subsequently reviewed by a radiation oncologist specializing in upper gastrointestinal malignancies. All patients received rotational volume-modulated radiotherapy by the linear accelerator with 6-MV photons. Any disagreements should be settled through consensus. GTV and GTV-ND were classified as primary tumors and lymph node involvement, respectively. The primary tumors and the involved nodes were identified using physical examination, endoscopic ultrasound, endoscopy, computed tomography scans and positron emission computed tomography. Furthermore, the CTV was defined as the primary tumor plus 3-cm superior and inferior expansion margins, as well as a 1-cm radial expansion margin. Prophylactic irradiation of the lymph node drainage area is performed in patients aged 50 to 79 years. Depending on whether the underlying tumor is in the upper or lower part of the esophagus, it may target lymph nodes in the abdomen or supraclavicular region. The planning target area was defined as a margin of 0.5–1 cm around the clinical tumor volume to account for tumor motion and setting changes in patients aged 80 years or older who were not eligible for prophylactic irradiation of lymph node drainage areas.

### Evaluation of treatment outcome

Two experienced radiation oncologists collaborated to assess the efficacy of the treatment using imaging examination within 3 months of the end of radiotherapy based on the efficacy evaluation criteria in the solid tumor criteria [21]. Responses are classified as CR (complete response, no residual tumor), PR (partial response, the longest diameter of the tumor was < 70% of its original size), PD (progressive disease, at least a 20% increase in the longest diameter of the tumor compared to the original size), and SD (stable disease, neither sufficient shrinkage for PR nor sufficient increase for PD). In this study, patients of CR or PR were defined as responders and those of SD or PD were non-responders. The positioning CT before CCRT was used in this study to predict responders and non-responders.

### Feature extraction

**Fig. 1:**
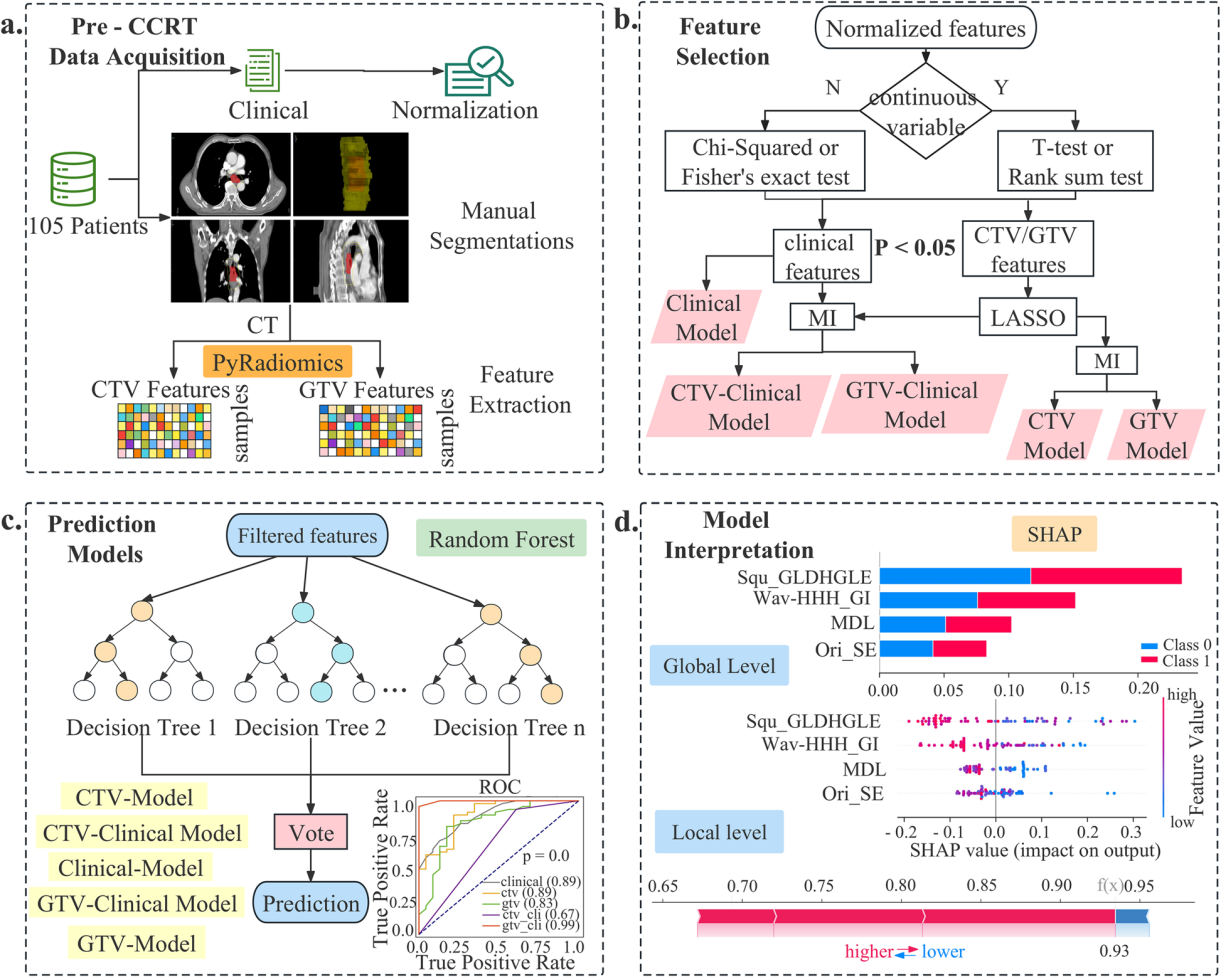
Overall pipeline of this work. **a** Pre-CCRT data acquisition and delineations. Cases were screened according to patient inclusion criteria. ROIs were outlined by experienced radio-oncologists. Features were extracted using PyRadiomics from ROIs. **b** Feature selection. Clinical, CTV and GTV features were filtered by statistical analysis, lasso and mutual information in sequence as described in the feature selection section. **c** Construction and comparison of five predictive models with random forest classifiers and filtered features including CTV, CTV-Clinical, Clinical, GTV and GTV-Clinical models. **d** Model interpretation at the global and local levels with SHAP algorithm

A significant variety of engineered hard-coded feature algorithms were integrated in the open source PyRadiomics python library for the high-throughput processing and extraction of putative image features from medical image data [22]. We applied PyRadiomics V3.0.1 to the CTV and GTV regions respectively (Fig. 1a) to extract the radiomics features, including first-order statistics, shape descriptors (2D and 3D), second-order and higher-order texture features. A fixed bin size of 25 bins was used for the intensity discretization process. No normalization was performed. The images were re-sampled spatially at 3 × 3×3mm with the help of the sitkBSpline interpolator. To assure non-negative integers when calculating first-order features in CT scans, 1000 is added to the grayscale intensity calculation for the energy, total energy, and root mean squared. Texture features include types of Gray Level Co-occurrence Matrix (GLCM), Gray Level Size Zone Matrix (GLSZM), Gray Level Run Length Matrix (GLRLM), Neighboring Gray Tone Difference Matrix (NGTDM) and Gray Level Dependence Matrix (GLDM). IBSI-compliant image features were calculated from raw and filtered CT images with Wavelet (8 combinations of wavelet decompositions from high- and low band-pass filters in x, y, z directions, respectively), Laplacian of Gaussian (LoG with σ of 1.0, 2.0, 3.0, 4.0, 5.0), Square, Square Root, Logarithmic, Exponential and Gradient, respectively by implementing PyRadiomics embedded filters [23]. The feature definitions and interpretations have been presented on this website (https://pyradiomics.readthedocs.io/en/latest/features.html) by pyradiomics community.

### Feature selection and signature construction

In order to avoid overfitting risks in our study, we performed feature selection on the training set by selecting a maximum number of features equal to the number of training sets divided by 20 (70/20≈4). The process of feature selection involved three steps (Fig. 1b). Firstly, statistical tests were conducted to keep features with statistical significance between responders and non-responders on the training dataset for the next selection step. Different statistical testing methods were employed depending on the type of variables analyzed. For continuous variables, Kolmogorov–Smirnov test (*kstest ()*) was used to test whether the data follows a normal distribution [24]. Subsequently, for normally distributed features, independent samples t-tests are conducted, while rank-sum tests are performed for non-normally distributed features. Categorical variables were subject to Chi-square test (*chi2_contingency()*) or Fisher's exact test (*fisher_exact()*)[24] as appropriate. Next, we utilized the least absolute shrinkage and selection operator (LASSO),*LassoCV()* with tenfold cross-validation from *sklearn* package, on the training set to reduce high feature dimensionality by shrinking the coefficients of all features and discarding the coefficients of irrelevant features to zero [25]. To avoid overfitting risks, the number of features was controlled to less than four using a mutual information (MI) method, *SelectKBest()*) from *sklearn* package [26]. It identifies the most relevant features related to the target variable among many features by measuring the degree of mutual information between the input features and the target variables (responders versus non-responders) on training dataset. The strongest ranked features based on mutual information were selected as the final feature set.

Figure 1b shows the signature construction and feature selection process for each model. Specifically, features for the Clinical-model were extracted from clinically relevant information followed by statistical tests. For the Radiomics-models, features were extracted from radiomics data obtained from the CTV and GTV regions, respectively. Final features were selected with LASSO and MI. To construct the radiomics-clinical model, statistically significant differential clinical features and LASSO selected differential radiomics features were combined, and then further selected with MI.

To evaluate the  reproducibility and robustness of the radiomics features chosen in our model, we repeated the feature selection procedure for 500 rounds with bootstrapping, and ranked the frequencies of all selected features from all rounds. The assumption is that if our selected features are  reproducible and robust, our selected features should be presented on the top of the list. Overall, in all statistical analyses conducted, we deemed *P* < 0.05 to be statistically significant and performed all calculations using Python.

### Model construction and comparison

Random forest is an integrated algorithm of decision trees and belongs to the Bagging ensembles (Fig. 1c) [27]. Random forest has high accuracy, generalization performance, and stability by voting of multiple weak classifiers. Training and testing sets were split by stratified sampling. Random forest machine learning models was constructed to predict radiotherapy efficacy for ESCC on the training set (*n* = 70) based on the constructed radiomics signatures. Depending on the input features, five different models were constructed in our study, i.e., Clinical model (clinical feature only), CTV model (radiomics features extracted from CTV region), GTV model (radiomics features extracted from GTV region), CTV-Clinical model (radiomics features extracted from CTV region plus clinical feature) and GTV-Clinical model (radiomics features extracted from GTV region plus clinical feature). Model performance was compared by AUC, accuracy, precision, recall and F1-Score index on the testing set (*n* = 24). Since AUC can synthesize a classifier's classification effect at various thresholds without regard to a single threshold value, it is typically regarded as one of the key metrics for comparing classifier performance. We utilized bootstrap approach to calculate confidence intervals and the Friedman rank test, which can reduce the influence of random errors and thus more objectively measure the variability and stability of classifier performance. The recall, true positive rate (TPR), false positive rate (FPR), precision, accuracy and F1-Score were calculated as follows:

1 $$\mathrm{TPR}=\mathrm{Recall}=\mathrm{TP}/\left(\mathrm{TP}+\mathrm{FN}\right),$$

2 $$FPR=FP/\left(FP+TN\right),$$

3 $$Precision=TP/\left(TP+FP\right),$$

4 $$Accuracy=TP+TN/TP+TN+FP+FN,$$

5 $$F1-Score=PE \times TPR/\left(PE+TPR\right) \times 2,$$

### Model interpretability with SHapley Additive exPlanation (SHAP)

SHAP (SHapley Additive exPlanation), a Python-based model interpretation package, can explain the results of any machine learning model (Fig. 1d) [28, 29]. Before attempting to interpret the model at the global and local levels, it is necessary for SHAP to calculate the marginal contribution of features. In SHAP, the cooperative game theory serves as the inspiration for the additive explanatory model. The SHAP value (*f(x)*) of each sample is finally depicted as the additive outcome of each feature's contribution ($\phi_{i}$). All features are regarded as contributors. Each sample receives a predictive value from the model, which is linearly added to the contribution value of each feature (Eq. 7). The calculation of feature-to-feature interaction effects (Eq. 8), where the interaction effect is the joint contribution of each pair of features with all other features and is calculated with TreeSHAP for tree-structured models (for example, random forests).

6 $$f(x)=\phi_0+\sum_{i=1}^M\phi_i,$$

7 $$\phi_{i} (f,x) = \sum\limits_{s^{\prime} \subseteq x^{\prime}} {\frac{|s^{\prime}|!(M - |s^{\prime}| - 1)!}{{M!}}} [f_{x} (s^{\prime}) - f_{x} (s^{\prime}\backslash i)],$$

8 $$\begin{gathered} \phi_{j,k} = \sum\limits_{{s^{\prime} \subseteq \backslash \{ j,k\} }} {\frac{|s^{\prime}|(M - |s^{\prime}| - 1)!}{{2(M - 1)!}}\delta_{j,k} (s^{\prime}),(j \ne k)}, \\ and,\delta_{j,k} (s^{\prime}) = f_{x} (s^{\prime} \cup \{ j,k\} ) - f_{x} (s^{\prime} \cup \{ j\} ) - f_{x} (s^{\prime} \cup \{ k\} ) - f_{x} (s^{\prime}) \\ \end{gathered}$$

The SHAP value *f(x)* of each sample *x* is calculated from all constants $\phi_{0}$ plus the contribution $\phi_{i}$ of each feature *i* over all the *M* features of that sample. The contribution $\phi_{i}$ of feature *i* is calculated by adding the shapley values $f_{x} (s^{\prime}) - f_{x} (s^{\prime}\backslash i)$. of all possible feature combinations of different orders and then weighting the sum, where $s^{\prime}$ is the subset of features used in the model, *x* is the feature vector representing a sample to be interpreted, and *M* is the total number of features. The weight of each feature combination among all possibilities would be expressed as $\frac{\left|s'\right|!\left(M-\left|s'\right|-1\right)!}{M!}$. The interaction effect between features *i* and *j* is quantified by subtracting each feature's primary effect from their total effects (Eq. 8).

## Results

### Patient clinical statistics

One hundred and five participants were enrolled in the study (Table 1) including 87 men and 18 women. The average age was 67.28 years (41—82 years). Patients with squamous cell esophageal cancer in clinical stages II, III and IVA were 29, 63 and 13, respectively. Initial CA72-4, CEA, and CA19-9 levels as tumor markers for esophageal carcinoma were present in 95% of ESCC patients, while initial CYFRA21-1 and SCC levels were known in 91% of patients. Missing values were filled with the median of the training set, testing set and validation set respectively.

**Table 1:** Basic patient information

Clinical feature	Category	N / Mean ± Std
Sex	Male	87
	Female	18
Age (year)		67.28 ± 8.57
BMI (kg/m2)		22.40 ± 3.11
BSA (m2)		1.62 ± 0.15
HGB (g/l)		125.29 ± 16.89
CA72-4 (IU/ml)		8.20 ± 22.20
CYFRA21-1 (ng/ml)		9.09 ± 29.39
SCC (ng/ml)		4.08 ± 9.79
CEA (ng/ml)		2.83 ± 2.02
CA19-9 (U/ml)		14.76 ± 33.24
LL(cm)		7.68 ± 2.81
MDL(cm)		3.60 ± 1.23
T stage	T1-2	20
	T3	74
	T4	11
N stage	N0	25
	N1	25
	N2-3	55
M stage	M0	105
TNM Stage	II	29
	III	63
	IVA	13

*N* The number of patients in each group, *Mean,* Mean of the feature values in the group, *Std* Standard deviation of the group's feature value*s*, *BMI* Body Mass Index, *BSA* Body Surface Area, *HGB* Hemoglobin, *CA72-4* Carbohydrate Antigen 72–4, *CYFRA21-1* Cytokeratin Fragment 21–1, *SCC* Squamous Cell Carcinoma antigen, *CEA* Carcino-embryonic Antigen, *CA19-9* Carbohydrate Antigen 19–9, *LL* Lesion Length, *MDL* Maximum Diameter of the Lesion

### Feature selection for clinical features as input into clinical-model

Table 2 shows the distribution of the patients in response vs non-response in the training and test datasets. The student’s *t-test* or rank sum test (continuous variables) and the chi-square test or Fisher's exact test (categorical variables) were used for variance analysis (Fig. 1b, Table 2). Table 2 demonstrates that the only statistically significant variable was MDL (*p* < 0.05) in response vs non-response groups in the training, testing and validation sets. The remaining clinical variables were all non-significant (*p* > 0.05). Thus MDL is the only feature input into the following Clinical Model.

**Table 2:** Statistical analysis of clinical features

Feature	Category	Training set (N / Mean ± Std)			Testing set (N / Mean ± Std)			Validation set (N / Mean ± Std)		
		Response (*n* = 47)	Non-response (*n* = 23)	*P* -value	Response (*n* = 16)	Non-response (*n* = 8)	*P*-value	Response (*n* = 4)	Non-response (*n* = 7)	*P* -value
Sex				1			1			0.98
	Male	38	19		14	8		2	6	
	Female	9	4		2	0		2	1	
T stage				0.05						
	T1-2	5	7		5	0	0.12	0	3	0.3
	T3	37	16		7	7		3	4	
	T4	5	0		4	1		1	0	
N stage				0.88						
	N0	12	7		2	1	0.84	0	3	0.46
	N1	10	4		6	2		2	1	
	N2-3	25	12		8	5		2	3	
M stage	M0	47	23	1	16	8	1	4	7	1
TNM Stage				0.27			0.26			0.53
	II	14	8		4	0		0	0	
	III	28	15		7	6		3	4	
	IVA	5	0		5	2		1	3	
Age (year)		67.45 ± 9.01	66.17 ± 8.09	0.27	66.75 ± 8.01	70.75 ± 6.27	0.20	67.25 ± 12.92	67.00 ± 8.00	0.97
BMI (kg/m2)		22.30 ± 3.08	22.94 ± 3.52	0.45	21.12 ± 2.40	22.32 ± 3.36	0.48	25.24 ± 1.99	22.73 ± 3.06	0.92
BSA (m2)		1.6 ± 0.14	1.63 ± 0.18	0.66	1.61 ± 0.14	1.58 ± 0.13	0.44	1.64 ± 0.14	1.65 ± 0.19	0.22
HGB (g/l)		128.28 ± 14.62	126.78 ± 16.56	0.71	121.75 ± 17.43	116.13 ± 22.50	0.43	126.28 ± 22.50	118.29 ± 20.36	0.85
CA72-4 (IU/ml)		4.91 ± 9.45	16.80 ± 39.17	0.29	2.58 ± 2.37	3.06 ± 2.48	0.54	7.53 ± 8.29	21.85 ± 38.32	0.40
CYFRA21-1 (ng/ml)		7.07 ± 6.20	18.80 ± 59.84	0.24	5.49 ± 3.02	5.19 ± 5.04	0.26	4.68 ± 2.02	6.46 ± 4.75	0.71
SCC (ng/ml)		5.25 ± 14.33	2.56 ± 1.52	0.52	4.11 ± 3.94	3.83 ± 3.59	0.95	2.51 ± 0.53	2.18 ± 0.49	0.85
CEA (ng/ml)		2.91 ± 2.56	2.96 ± 1.46	0.25	2.25 ± 0.91	2.69 ± 1.10	0.58	2.77 ± 1.18	3.35 ± 2.60	0.45
CA19-9 (U/ml)		13.01 ± 13.18	12.19 ± 8.67	0.56	29.78 ± 81.64	10.48 ± 7.19	0.93	14.68 ± 8.63	5.24 ± 2.52	0.06
LL(cm)		7.74 ± 2.69	2.76 ± 2.17	0.14	9.13 ± 3.52	7.06 ± 2.76	0.23	7.88 ± 2.60	7.50 ± 3.37	0.57
MDL(cm)		3.76 ± 1.25	2.95 ± 0.86	0.01	4.51 ± 1.10	3.11 ± 1.18	0.02	4.16 ± 0.86	2.61 ± 0.82	0.04

### Selected radiomics features

**Fig. 2:**
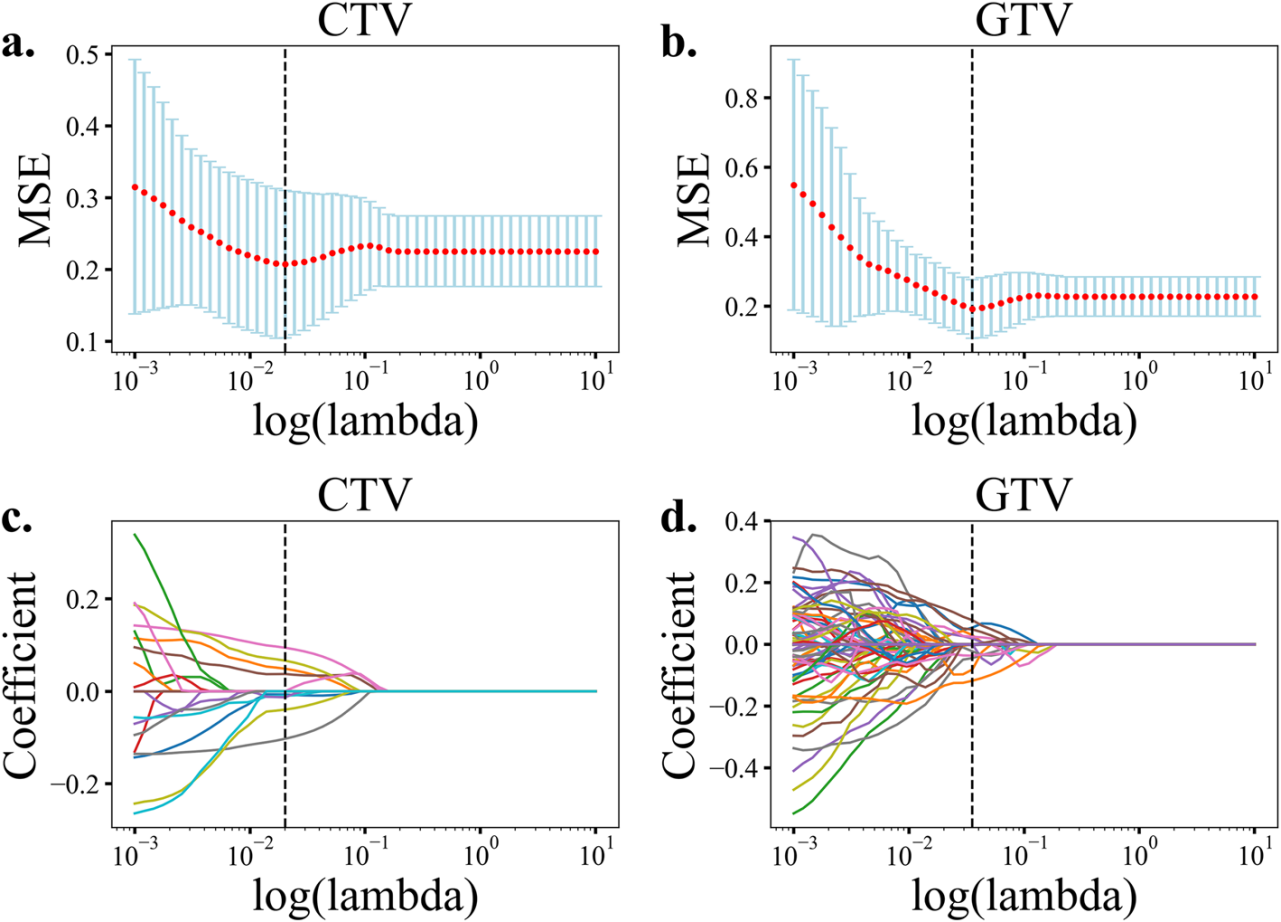
Lasso feature filtering. Selection of the best penalty coefficient value (lambda) that has the minimum mean squared error (red dot) (MSE) in tenfold cross-validation of LASSO regression for CTV (**a**) and GTV (**b**) features. The optimal lamda value (CTV: 0.02, GTV:0.04, dashed vertical line) was chosen as the smallest mean MSE of the training sample. Lasso coefficient profiles (**c**, **d**) against lambda value and the best lambda value (dashed vertical line) where retained features had non-zero coefficients

One thousand seven hundred and sixty-two radiomics features were extracted from the original image including 14 shape features, 18 first-order statistics features times 19 (equal to 342), and 74 texture features times 19 (equal to1406) of one original and 18 wavelet or Laplacian of Gaussian filtered images (equal to 19 images). The 74 texture features include 23 GLCM, 16 GLRLM, 5 NGTDM, 14 GLDM and 16 GLSZM features. To filter the features for CTV and GTV, two steps were taken including differential statistical analysis and lasso feature reduction for CTV model and GTV model between responders vs. non-responders. All feature selections were based on training set (Fig. 1b). 20 CTV features and 455 GTV features remained after t-test or rank sum test was applied to the 1762 Pyradiomics extracted features. After lasso dimensionality reduction (Fig. 2) on the statistically filtered features, 9 features remained for CTV and 12 remained for GTV, respectively. To avoid overfitting, at most four features are eventually left respectively using the MI method. Table 3 shows the remaining features. As stated in our methodology, following 500 rounds of bootstrap resampling and selected feature frequency ranking, we observed that our final selected features consistently ranked among the top ten most frequently selected ones (Table 4). This result demonstrates the robustness and reproducibility of our feature selection strategy.

**Table 3:** The final selected features as respective inputs into the five models

Model	Feature (Filter _Feature Class_Feature)	Abbreviation
Clinical	Maximum Diameter of the Lesion	MDL
CTV	wavelet-HLH_firstorder_Mean	Wav-HLH_FMean
	wavelet-LLL_glcm_Imc1	Wav-LLL_GI
	square_glcm_InverseVariance	Squ_GIV
	gradient_firstorder_10Percentile	Gra_F10P
CTV-Clinical	wavelet-LLL_glcm_Imc1	Wav-LLL_GI
	wavelet-HLH_firstorder_Mean	Wav-HLH_FMean
	square_glcm_InverseVariance	Squ_GIV
	Maximum diameter of the lesions	MDL
GTV	original_shape_Elongation	Ori_SE
	original _shape_Flatness	Ori_SF
	wavelet-HHH_glcm_Imc1	Wav-HHH_GI
	square_gldm_LargeDependenceHighGrayLevelEmphasis	Squ_GLDHGLE
GTV-Clinical	wavelet-HHH_glcm_Imc1	Wav-HHH_GI
	original_shape_Elongation	Ori_SE
	square_gldm_LargeDependenceHighGrayLevelEmphasis	Squ_GLDHGLE
	Maximum diameter of the lesions	MDL

**Table 4:** Rank of the top radiomics features in frequency through sample bootstrapping

Rank	Radiomics features	Frequency
CTV		
1	wavelet-HLH_firstorder_Mean	247
2	original_shape_MajorAxisLength	98
3	wavelet-LLL_glcm_Imc1	92
4	square_glcm_InverseVariance	90
5	wavelet-HLH_firstorder_Median	84
6	log-sigma-1–0-mm-3D_gldm_DependenceVariance	72
7	wavelet-HHH_firstorder_Skewness	63
8	wavelet-LHH_gldm_DependenceVariance	57
9	gradient_firstorder_10Percentile	51
10	squareroot_glrlm_LongRunLowGrayLevelEmphasis	39
GTV		
1	wavelet-LHH_gldm_LargeDependenceHighGrayLevelEmphasis	151
2	original_shape_Elongation	112
3	logarithm_gldm_LargeDependenceHighGrayLevelEmphasis	95
4	square_gldm_LargeDependenceHighGrayLevelEmphasis	92
5	gradient_glszm_SizeZoneNonUniformityNormalized	65
6	wavelet-HHH_glcm_Imc1	63
7	gradient_firstorder_Minimum	59
8	wavelet-LHL_ngtdm_Contrast	55
9	original_shape_Flatness	45
10	wavelet-HHL_ngtdm_Strength	45

### Features input into the CTV-Clinical and GTV-Clinical Models

The mutual information method was used to combine imaging histological features and clinical factors by calculating the interdependence between filtered features (CTV, GTV and clinical features) and response to CCRT (Fig. 1b). 3 radiomics features and one clinical feature in CTV-Clinical and GTV-Clinical model were included in the top four-feature matrices (Table 3), respectively. The three radiomics features include original shape elongation and texture features of wavelet HHH and square filtered images. The only clinical feature selected in GTV-Clinical Model and CTV-Clinical Model was MDL.

### Construction and comparison of the five models

**Fig. 3:**
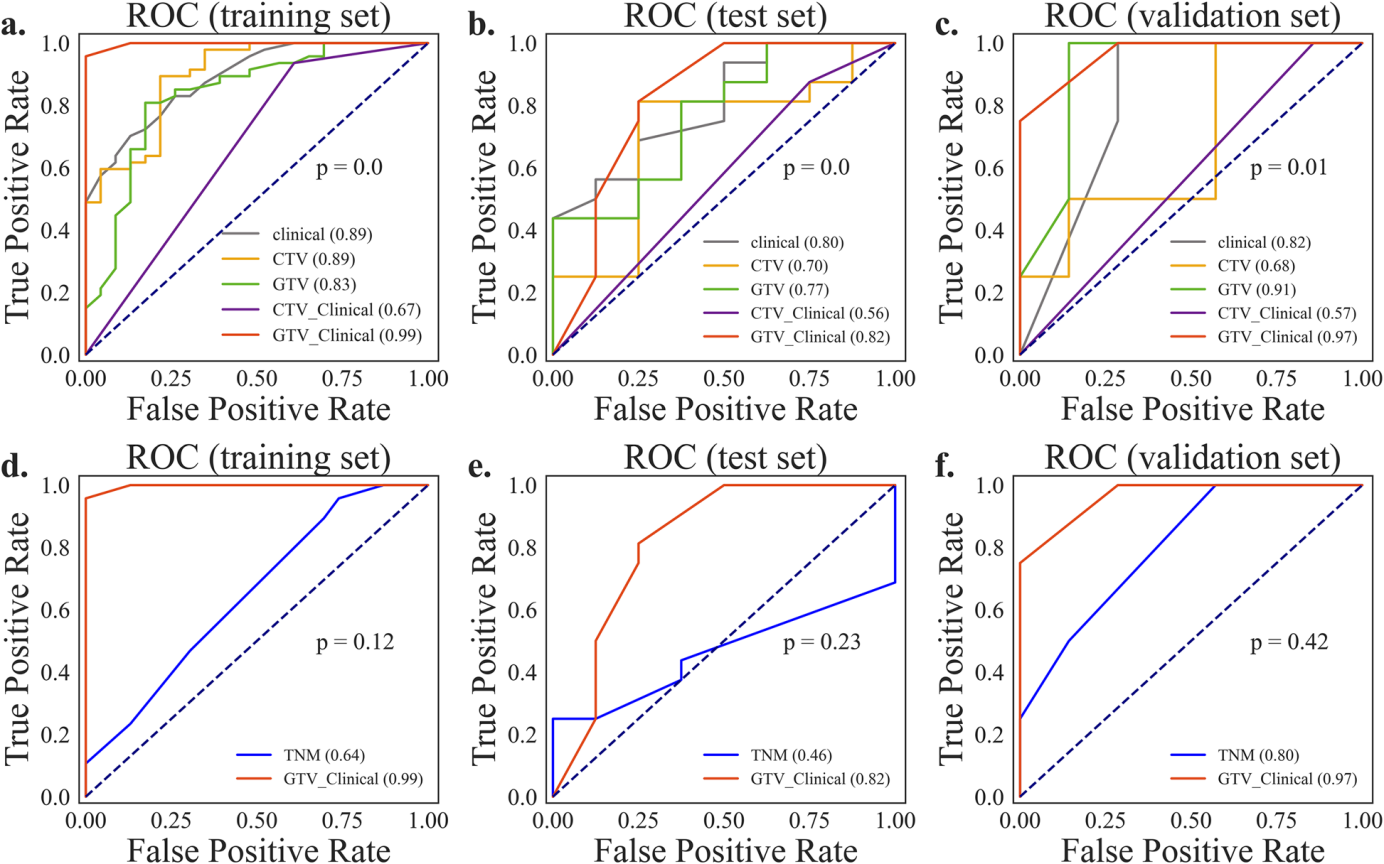
Comparison of the five model performance by *AUC* of ROC. *AUC*, Area under Receiver Operating Characteristic Curve). The five models include clinical, CTV, GTV, CTV-Clinical, and GTV-Clinical. Receiver Operating Characteristic (ROC) curve plots of training set (**a**), testing set (**b**) and validation set (**c**) and the comparison of the GTV-clinical model and the TNM model (**d**-**f**) illustrates the relationship between FPR and TPR (taking both positive and negative examples into account (Eq. 1 and 2)) and is used to assess the overall performance of the classifier

To build the Clinical model, CTV model, GTV model, CTV-Clinical model and GTV-Clinical model with training sets respectively, the features from Table 3 were loaded into the random forest classifier. Table 5 shows the tunned parameters of each model after the grid search method (*GridSearchCV()*) from sklearn package. After the model has been trained using the training set, a superior model is chosen by contrasting its scores using the testing set, and the model's generalization performance was confirmed using the validation set. As mentioned in method, results showed significant differences between the five models (Fig. 3a-c, p < 0.05). The confidence level of these differences was further verified by applying 1000 bootstrap resampling experiments to obtain confidence intervals for the AUC values of each model. At 95% confidence level, GTV-Clinical model has the largest AUC value and AUC value confidence interval, indicating that GTV-Clinical model is superior in classification performance on the dataset (Table 6). As the result displayed, an AUC of 0.82 and 95% confidence interval (CI) of 0.76–0.99 of GTV-Clinical on testing set and an AUC of 0.97 and 95% confidence interval of 0.84–1.0 on validation set are higher than those of other models (Table 6, Fig. 3a-c). The Clinical, CTV, GTV, and CTV-Clinical models all predicted CCRT responses significantly better than randomized guesses.

**Table 5:** Parameters of the random forest models

Model	n_estimators^a^	max_depth^b^	max_features^c^	random_state^d^
Clinical	6	5	1	2023
TNM	7	3	2	2023
CTV	126	2	1	2023
GTV	17	1	2	2023
CTV-Clinical	1	1	1	2023
GTV-Clinical	7	8	1	2023

^a^n_estimators, the number of trees in the forests

^b^max_depth, the maximum depth of the tree

^c^max_features, the number of features to consider when looking for the best split

^d^random_state, random state instance to control the reproducibility of the bootstrapping of samples and features

**Table 6:** Model performance comparison

Model	AUC (95% CI)	Accuracy	Precision	Recall	F1-Score
**training set:**					
Clinical	0.89 (0.81–0.95)	0.81	0.80	0.96	0.88
TNM	0.46(0.25–0.68)	0.73	0.73	0.96	0.83
CTV	0.89 (0.80–0.96)	0.83	0.81	0.99	0.88
GTV	0.83 (0.72–0.93)	0.76	0.73	1.0	0.85
CTV-Clinical	0.67 (0.56–0.78)	0.76	0.76	0.94	0.84
GTV-Clinical	0.99 (0.98–0.99)	0.96	0.94	1.0	0.97
**testing set:**					
Clinical	0.80 (0.60–0.96)	0.75	0.72	1.0	0.84
TNM	0.64 (0.51–0.77)	0.54	0.62	0.71	0.70
CTV	0.70(0.43–0.94)	0.63	0.67	0.88	0.76
GTV	0.77 (0.53–0.96)	0.75	0.75	0.94	0.83
CTV-Clinical	0.56 (0.4–0.75)	0.67	0.70	0.86	0.78
GTV-Clinical	0.82 (0.66–0.99)	0.71	0.74	0.88	0.80
**validation set:**					
Clinical	0.82 (0.57–1.0)	0.55	0.44	1.0	6.15
TNM	0.80 (0.50–1.0)	0.64	0.50	1.0	0.67
CTV	0.68 (0.25–1.0)	0.45	0.4	1.0	0.57
GTV	0.91 (0.67–1.0)	0.73	0.57	1.0	0.73
CTV-Clinical	0.57 (0.50–0.75)	0.54	0.44	1.0	0.62
GTV-Clinical	0.97 (0.84–1.0)	0.72	0.57	1.0	0.73

To further assess the performance of the resultant model on CCRT response prediction in real clinical context, we compared the GTV-Clinical model with the conventional TNM model, where the TNM model only considered T-stage and N-stage because all patients in our collection had the same M-stage of M0. Although the results of the Friedman rank test showed insignificant differences between the AUCs of the two models, a comparison of the 95% confidence intervals and the values of the AUCs showed that our model outperformed the TNM model (Table 6, Fig. 3d-f).

### Model interpretability with SHAP

**Fig. 4:**
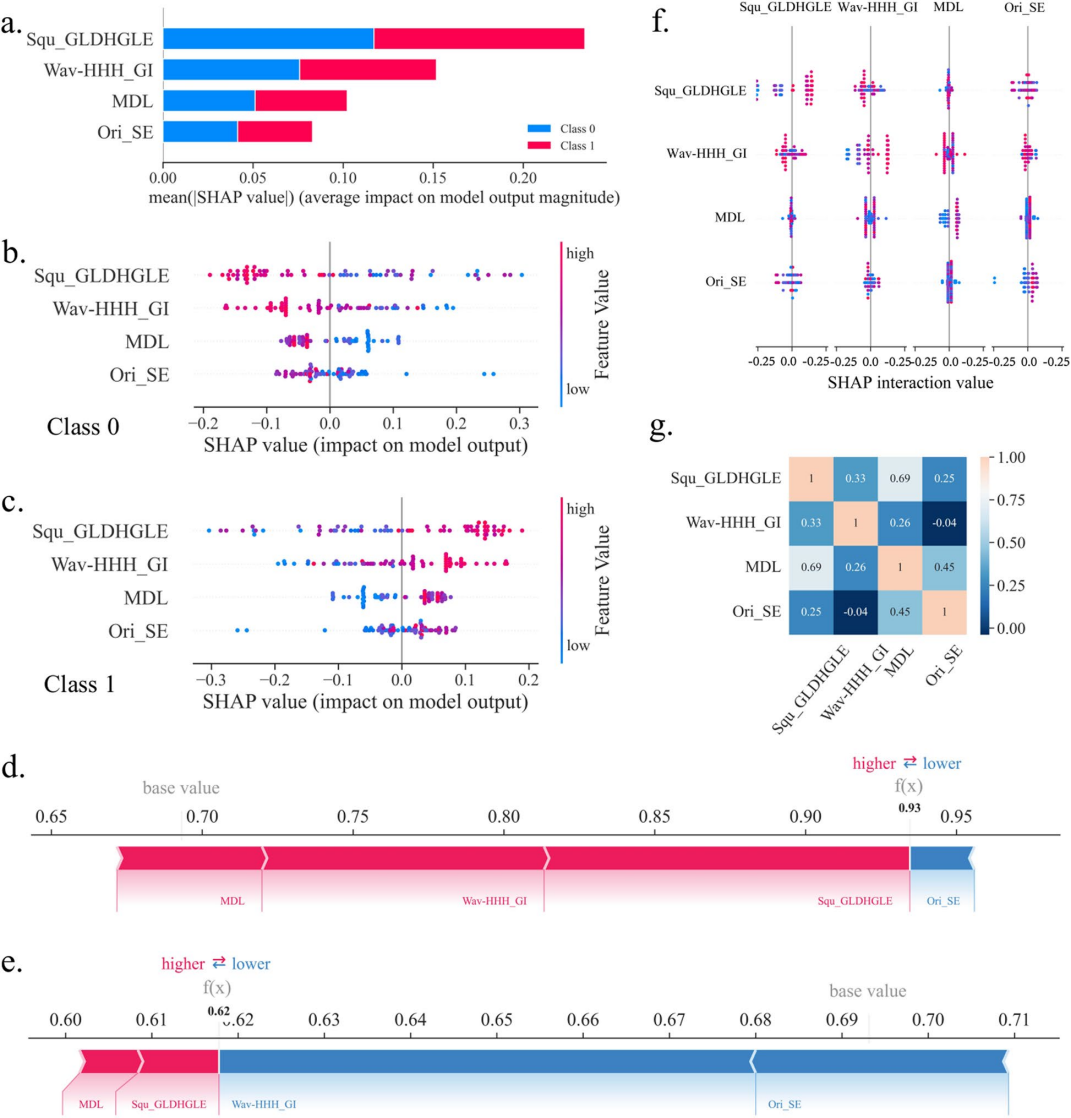
Model Interpretation by SHAP. **a** The overall contribution of each feature to the model prediction classification (class 0: non-response, class 1: response), in order of importance. **b** A detailed contribution to the class 0 model prediction, where the colors represent the magnitude of the feature values. **c** The detailed contribution to the class 1 model prediction. **d** Explanation of how features affect the model's predictions for a single sample, where the results show that this patient would be effectively relieved by performing CCRT. **e** An example that a model predicts that a patient will not achieve effective remission with CCRT. **f** Feature dependency visualization by interaction values between features. **g** Heat map of Pearson correlation coefficients between features

In the GTV-Clinical random forest model, the SHAP demonstrates how input features affect prediction outcomes at both the global and local levels. At the global level, the feature relevance and the contributions to the sample's positive and negative predictions are shown in the summary plot of Fig. 4a-c. Results show that both the first important feature Squ_GLDHGLE and the second important feature Wav-HHH_GI are texture features. The former, large dependence high gray level emphasis of GLDM with Square filter (Squ_GLDHGLE), reflects the texture’s homogeneity. And the latter, IMC1 feature of GLCM with Wavelet-HHH filter (Wav-HHH_GI), demonstrates the texture's complexity. Textural features enable quantification of information which is difficult to perceive by nude eyes, such as texture patterns and tumor tissue distribution [30, 31]. The elongation in the original image shape feature (original_shape_Elongation, Ori_SE) is the fourth predictive feature indicating whether the tumor's shape and edge are regular [31]. According to Fig. 4c, a positive correlation exists between the elevation of four features and the model output, indicating that higher values of these features are more likely to result in a prediction of class 1 (responder).

The SHAP force plot (Fig. 4d-e) allows for the analysis of individual samples at the local level and explains how the model predicts whether a patient will respond to treatment. The features in red increase output while the features in blue do the opposite. The CCRT prediction for this patient (Fig. 4d) is responsive when the output value of this sample in the model (*f(x)*: 0.93) is higher compared to the average output of all samples (base value: 0.69). Similarly, the non-responder could be forecasted in Fig. 4e, where *f(x)* < base value (0.62 < 0.69). By comparing the *f(x)* and base values for each sample, the force plot can help us understand how these model features predict the response of patients to CCRT before treatment. Our SHAP model indicates that higher values of all the four features in the final GTV-Clinical are beneficial to the CCRT response. The result indicates that high elongation index and MDL of the original CT image and high homogeneity and low complexity texture features of wavelet HHH and square filtered features respectively are beneficial to the CCRT response. The biological meaning of the texture features are intriguing and warrant further study at cellular and molecular level.

Feature dependencies could be checked by interaction values (Fig. 4f). When the interaction values of features do not clearly separate the two categories, there is no strong dependency between them (effective or ineffective). Measures of feature combination (such as polynomial features or FM of crossover features) must be taken into account if there are dependencies between them. Other than the features themselves (on the main diagonal), the combination of features does not classify well. Obviously, there is no multicollinearity problem among the features input to our model, which could be identified with the Pearson correlation coefficient heatmap (Fig. 4g).

## Discussion

Patients with esophageal cancer who are not suitable for surgery, are primarily treated with radical CCRT. Determining the sensitivity of patients to CCRT is a hot topic of clinical research. To predict the short-time efficacy, the response of esophageal squamous carcinoma to CCRT, we developed five models based on a random forest algorithms, tumor target areas and clinical parameters (Clinical model, CTV model, GTV model, CTV-Clinical model and GTV-Clinical model). We concluded that the GTV-Clinical model is superior to others in predicting the efficacy of CCRT in esophageal squamous cancer. The superior of the GTV-Clinical model to the classic TNM model suggests its potential utility in clinical diagnosis and treatment decisions (Table 6).

We proposed a pipeline for feature selection, i.e., statistical analysis, LASSO downscaling and Mutual Information were used in sequence to filter 13 clinical and 1762 CT image features extracted by Pyradiomics in CTV and GTV, respectively (Fig. 1b). Specifically, univariate statistical analysis techniques were used to filter the features according to their distribution characteristics. A penalty function was simultaneously constructed to provide a more appropriate model when features were estimated through LASSO. Only regression coefficients of features significantly other than zeros were considered in further feature selection to reduce the dimensionality of high-dimensional data. Finally, the mutual information method (MI), a non-parametric approach based on k-nearest neighbor distance entropy estimation, was employed to better integrate the clinical and radiomics features. Top 4 features with the highest correlation between features and response in the training dataset were then trained for modeling.

We concluded that the GTV-Clinical Model, which combined GTV and clinical features, has the best predictive performance for the response of esophageal squamous carcinoma to CCRT. To comprehend the influence of the eigenvalues on the model at both the global and local levels, the SHAP technique is used, which makes it easier to understand our model. The SHAP model can account for the influence of all features on the model (global), as well as the interaction effect of each feature on individual patient predictions (local). Interpreting our GTV-Clinical random forest model with SHAP increased the model interpretation, utilization and generalization, especially for clinicians. We found a higher elongation and MDL value and homogeneity Imc1 in wavelet HHH filtered image and low complexity of square filtered image are beneficial to CCRT response. The biological meaning of the result is very intriguing and warrant molecular and cellular characterization of the features.

It is hypothesized that intratumoral heterogeneity affects CCRT responsiveness [32, 33]. The heterogeneity of tumors may result in sampling errors through surgery and biopsy. These surgical or biopsy data should be cautiously used as a reference for modifying treatment before or during treatment because the only once sample is possibly biased before all preoperative treatments and procedures have been finished. Employing discovered radiomics biomarkers as a non-invasive approach will increase the prognostic accuracy of ESCC pre-treatment. The positioning CT prognostic biomarkers may shed light on a cancer’s phenotypes and even behind genotypes, which are invisible with nude eyes. These radiomics plus clinical markers obtained by our proposed feature selection method may assist in determining which patients would be unlikely to benefit from CCRT and would benefit more from alternate therapies or stringent follow-up schedules. It is crucial to define the primary tumor site before CCRT therapy of ESCC, and the GTV-clinical radiation model based on radiomics may help us identify the CCRT beneficiary population. The GTV-Clinical Model is superior to other models, even the CTV-clinical Model, in predicting the short-term efficacy of CCRT in ESCC, indicating that the primary tumor is better in evaluating the short-term efficacy of patients.

At the start of the experiment, we collected 13 clinical features for our investigation, which were selected based on domain knowledge and literature review. Studies have shown that indicators of nutritional status will affect the survival of ESCC patients [34]. And tumor clinical stage [35], maximum diameter of the lesion [36] and lesion length [37] may affect the efficacy and prognosis of esophageal squamous cell carcinoma. When filtering for 13 clinical features, we found that MDL was the only statistically significant biomarker on the training and testing datasets. It is consistent with previous study in which maximum pre-treatment esophageal wall thickness was independently associated with patient response to chemotherapy [38].

There are some limitations in this study. First, our study may have a patient selection bias. Patients may not have been strictly randomized, and all data came from a single center. Secondly, the sample size is insufficient. The method needs to be validated further based on external centers and large-scale sample data.. Finally, we compared the efficacy of CCRT in ESCC across five models, and found that the GTV of ESCC may play a clear role in CCRT's short-term efficacy. However, whether GTV-clinical model is most effective in predicting the patient overall survival in CCRT needs follow-up findings.

## Conclusion

The GTV-clinical model based on radiomics and clinical features outperforms other models, including the CTV region-based model and the classic TNM model, in predicting the short-term outcomes of concurrent chemoradiotherapy (CCRT) in patients with esophageal squamous carcinoma (ESCC). The model may help clinicians identify the patients who will benefit from CCRT and determine which ones are less likely to benefit but more likely to benefit from alternative therapies or rigorous follow-up programs. The proposed feature interpretation by SHAP model is intriguing and warrant further study on their association with spatial molecular and cellular properties of ESCC. However, this study has limitations, including patient selection bias, inadequate sample size, and the need for further validation with data from external centers and large samples. Future studies should investigate whether the GTV-clinical model is effective in predicting overall survival in patients with CCRT. Overall, this study highlights the potential of radiomics as a non-invasive approach to improve prognostic accuracy and improve treatment decisions prior to ESCC treatment.

## Data Availability

The feature datasets and codes generated during the current study are available in a publicly accessible repository (https://github.com/zyx-wu/Predict-CCRT-Response-in-ESCC).

## Abbreviations

NCCN: National comprehensive cancer network
CCRT: Concurrent chemoradiotherapy
SHAP: Shapley additive explanation
GTV: Gross tumor volume
CTV: Primary tumor and the uninvolved lymph nodes at risk of microscopic disease
ROI: Regions of interest
LASSO: Least absolute shrinkage and selection operator
MI: Mutual Information
CI: Confidence Interval
MDL: Maximum diameter of lesion
CR: Complete response
PR: Partial response
PD: Progressive disease
SD: Stable disease
GLCM: Gray level co-occurrence matrix
GLSZM: Gray level size zone matrix
GLRLM: Gray level run length matrix
NGTDM: Neighboring gray tone difference matrix
GLDM: Gray level dependence matrix
AUC: Area under receiver operating characteristic curve
